# Network-Based Pharmacology and Bioinformatics Study on the Mechanism of Action of Gujiansan in the Treatment of Steroid-Induced Avascular Necrosis of the Femoral Head

**DOI:** 10.1155/2022/8080679

**Published:** 2022-07-23

**Authors:** Guo-wu Ren, Shuai-bo Wen, Jie Han, Zhi-wei Xu, Wen Qi, Yu-zhi Shang, Yu-kun Wu

**Affiliations:** ^1^Guangxi University of Traditional Chinese Medicine, Nanning City, Guangxi Zhuang Autonomous Region 530001, China; ^2^Ruikang Hospital Affiliated to Guangxi University of Traditional Chinese Medicine, Nanning City, Guangxi Zhuang Autonomous Region 530011, China

## Abstract

**Objective:**

To investigate the main pharmacological basis and mechanism of action of Gujiansan in the treatment of steroid-induced avascular necrosis of the femoral head (SANFH).

**Methods:**

The active constituents and targets of Gujiansan were screened by using TCMSP and other databases, and relevant disease targets were obtained by analyzing the microarray of SANFH in the GEO database. The intersection of the two was taken to obtain the potential targets of Gujiansan for the treatment of SANFH, and key active constituents were screened with the “active constituent-target” network constructed by the Cytoscape software; then, the STRING database was used to construct the protein interaction network to screen the key targets. The Gene Ontology and Kyoto Encyclopedia of Genes and Genomes enrichment analyses of key targets were performed by the DAVID database, and the relationship between the “key active constituent-key target-key signaling pathway” was explored. Finally, the molecular docking between key active constituents and key targets was verified. In addition, qPCR detection technology was used to evaluate the preventive and therapeutic effects of key active constituents of Gujiansan in a rat osteoblast model of SANFH to verify the possible mechanism of the effect of Gujiansan in the treatment of SANFH.

**Results:**

(1) 106 active constituents and 55 targets were obtained for the treatment of SANFH. (2) Quercetin, luteolin, kaempferol, cryptotanshinone, and naringenin were the key active constituents for the treatment of SANFH. (3) IL1B, STAT3, CAT, PTGS2, and MAPK3 were the key targets for the treatment of SANFH. (4) IL1B, STAT3, CAT, PTGS2, MAPK3, and HMOX1 are key targets in the protein interaction network. (5) DAVID enrichment analysis mainly covers the regulation of DNA-binding transcription factor activity, positive regulation of cytokine production, and response to oxidative stress and other biological processes, involving IL-17, AGE-RAGE, C-type lectin receptor, and other signaling pathways. (6) Gujiansan is a multitarget and multisignaling pathway for the treatment of SANFH. (7) Good binding activity exists between key active constituents and key targets.

**Conclusion:**

This study analyzes the potential mechanism of action of Gujiansan in the treatment of SANFH with network pharmacology, which can provide a reference for the further study of its pharmacological basis and targets.

## 1. Introduction

Avascular necrosis of the femoral head (ANFH), also known as osteonecrosis of the femoral head (ONFH), is a disabling disease that occurs in young and middle-aged people. According to statistics, the total number of diagnosed patients worldwide has exceeded 30 million, including more than 8 million in China, and about 75,000 to 150,000 new cases are added every year [1, 2]. The etiology of ONFH includes traumatic and nontraumatic causes. Traumatic ONFH is usually caused by traumatic factors of fractures, dislocations, and contusions. The pathogenesis of nontraumatic ONFH is not well understood, and the main risk factors include corticosteroid use, alcohol abuse, and autoimmune disease. Steroid-induced avascular necrosis of the femoral head (SANFH) is the most common type of nontraumatic ANFH, and 47.4% of nontraumatic ANFH was directly related to steroid hormone abuse as suggested in an epidemiological survey of ANFH in Japan [3]. The main pathogenetic theories are the theory of disorder of lipid metabolism, the theory of reduced osteogenic potential of bone marrow mesenchymal stem cells, the theory of impaired blood supply, the theory of inflammation and apoptosis, the theory of gene polymorphism and noncoding RNA, etc. [4]. SANFH develops rapidly and can progress to femoral head collapse, necrosis, or even severe hip arthritis and loss of hip mobility within a few years, which seriously affects people's quality of life. The clinical treatment of terminal SANFH is mainly surgery, but it cannot delay or reverse the progression of SANFH, and there are difficulties in rehabilitation, secondary revision of the joint prosthesis, and heavy financial burdens [5, 6].

Traditional Chinese medicine (TCM) has been widely used in the treatment of orthopedic diseases (such as SANFH, osteoporosis, KOA, and gout) in Japan, Korea, China, and other Asian countries and regions. It is gradually accepted in clinical applications and medical institutes worldwide [7–10]. Numerous basic and clinical experiments show that compound TCM is economical and has multiplicity and low side effects in treating SANFH [10, 11]. Gujiansan is a formula created by Professor Wei Guikang, a master of TCM, based on the theory of traditional Chinese therapeutics and medication as well as his accumulated experience in the past decades. Gujiansan consists of Radix Panacis Quinquefolii, Heterophylly Falsestarwort Root, Radix Astragali, Cornu Cervi Pantotrichum, Notoginseng, Rhizoma Drynariae, Ganoderma, Radix Salviae Miltiorrhizae, Moghania, Litsea, Endothelium Corneum Gigeriae Galli, Herba Asari, and Saffron. Previous studies by the team indicate that Gujiansan acts as a vascular and bone protector by promoting the levels of HIF-1*α*, autophagy-associated factors (including BNIP3, LC3, and Beclin-1), and bone formation markers (including osteocalcin (OC), bone-specific alkaline phosphatase (BAP)) and decreasing the levels of proinflammatory cytokines (including TNF-*α*, IL-6, and CRP) [12].

**Figure 1:**
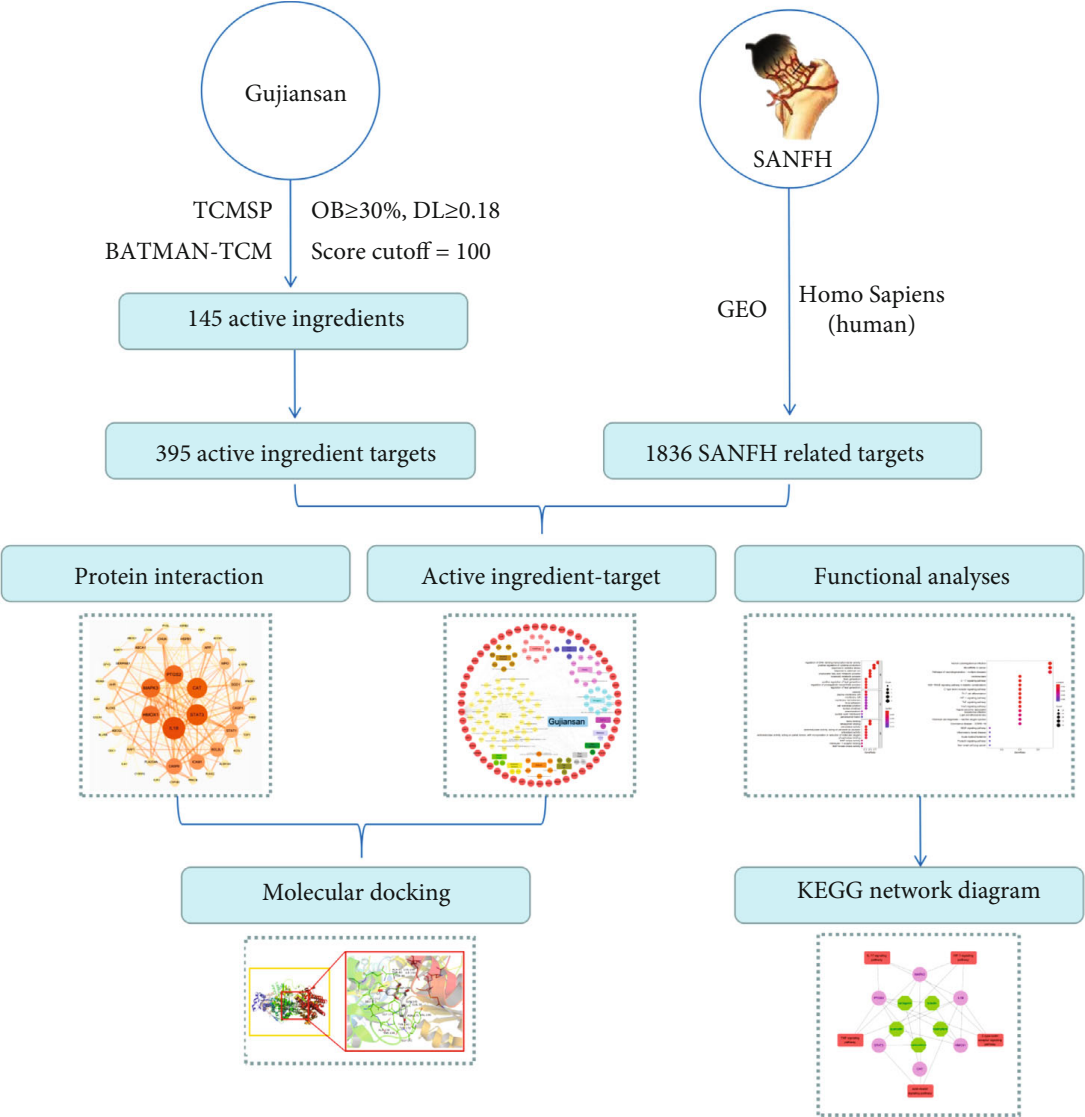
Network pharmacology flow chart of the mechanism of action of Gujiansan in the treatment of SANFH.

However, due to the complex multicomponent, multitarget, and multipathway intervention of herbal formulas for SANFH treatment, the mechanism of action of Gujiansan on this disease has not been fully elucidated and remains to be further investigated. Currently, emerging network pharmacology and bioinformatics technologies are widely used in pharmacology research [13, 14]. Therefore, based on network pharmacology and molecular docking technology, this study analyzes the molecular mechanism of Gujiansan in the treatment of SANFH from a microscopic perspective and in vitro experiments with the aim of providing a direction and reference for the further study of the basic pharmacology of Gujiansan in the treatment of SANFH (see Figure 1 for the research process).

## 2. Data and Methods

### 2.1. Components of Gujiansan and Target Mining

In this study, TCMSP and BATMAN-TCM databases were used to extract and retrieve the chemical components of each Chinese medicine herb in Gujiansan (Radix Panacis Quinquefolii, Heterophylly Falsestarwort Root, Radix Astragali, Cornu Cervi Pantotrichum, Notoginseng, Rhizoma Drynariae, Ganoderma, Radix Salviae Miltiorrhizae, Moghania, Litsea, Endothelium Corneum Gigeriae Galli, Herba Asari, Saffron, and Rhizoma Smilacis Glabrae). Oral bioavailability (OB) is one of the most important pharmacokinetic parameters in drug absorption, distribution, metabolism, and excretion (ADME) as it indicates how fast and to what degree the effective components or active groups of oral drugs enter into the systemic circulation and can be absorbed. A higher OB value generally indicates better druglikeness (DL) of the bioactive molecules of a drug [15]. Therefore, OB ≥ 30% and DL ≥ 0.18 were set to screen the chemical components obtained in the TCMSP database and the corresponding target proteins were found. Then, the UniProt database was used to query the gene names corresponding to the target proteins. At the same time, set the threshold “score cutoff = 100” based on the principle of “component-target similarity” in the BATMAN-TCM database [16] to obtain the target and gene name corresponding to the chemical components of Chinese medicine. The results from the two databases were combined, and the duplicates were deleted to get the active constituent-target of Gujiansan. The involved database and related analysis platform in the study are shown in Supplementary Materials (including Table 5 and Table 6).

### 2.2. Collection of SANFH-Related Targets

Using the keywords “steroid-induced osteonecrosis of the femoral head,” “Human,” and “Peripheral blood” in the GEO public database [17], we retrieved the related chips of SANFH and obtained the matrix file GSE123568 and gene annotation file GPL15207. The chip contained 40 peripheral serum samples, including 30 patients with SANFH and 10 healthy controls. We reannotated the data with Perl language and corrected and classified it with R language. The limma package was used for the difference analysis of genes, *P* < 0.05 and |log_2_*FC*| ≥ 0.5 [18] were set as the filtering conditions to screen for the different genes, and the pheatmap package was used to draw the heat map of the different genes. The obtained results were the relevant targets of SANFH.

### 2.3. Prediction of Potential Targets and Construction of the “Active Constituent-Target” Network

The active constituents-targets of Gujiansan and the related targets of SANFH were uploaded to the Venny platform for mapping and intersection to obtain the potential targets of Gujiansan in the treatment of SANFH. The network diagram of its “active constituents-targets” was drawn by Cytoscape software, and the key active constituents were screened out according to the degree value.

### 2.4. Protein Interaction Network Construction

Cell life depends on the complex functional network between biological molecules. In this network, the protein-protein interaction is critical due to its versatility, specificity, and adaptability. The STRING database integrates all known and predicted protein associations, including physical interactions and functional associations [19]. In order to further study the mechanism of Gujiansan in the treatment of SANFH, the targets were imported into the STRING database, with the research species limited to “Homo sapiens,” and the connection score was set to >0.4 to obtain the protein-protein interaction relationship. The results were imported into Cytoscape, and “NetworkAnalyzer” was used for visual processing to construct a protein-protein interaction (PPI) network. “cytoHubba” [20] was used to screen the key targets of Gujiansan for the treatment of SANFH according to the degree value.

### 2.5. DAVID Enrichment Analysis

DAVID integrates biological data analysis to complete annotation of gene expression data, as well as Gene Ontology (GO) and Kyoto Encyclopedia of Genes and Genomes (KEGG) pathway enrichment analyses [21]. The DAVID database was used for GO functional enrichment analysis of key targets to study the biological function of Gujiansan in the treatment of SANFH. The KEGG pathway enrichment analysis was performed on key targets to study the signaling pathway of Gujiansan in the treatment of SANFH, and the bubble diagrams for GO and KEGG enrichment analyses were drawn with the ggplot2 package in R language.

### 2.6. KEGG Relation Network Construction

According to the signal pathways obtained, the key targets of key active constituents and their signal pathways were analyzed to obtain the relationship network of “key active constituents-key targets-key signal pathways.” The network was imported into Cytoscape software, and the first five key signal pathways were selected to draw the KEGG relation network diagram.

### 2.7. Molecular Docking Verification

In order to explore the interaction between the key active constituents and the key targets of Gujiansan in treating SANFH, molecular docking verification was carried out. ChemOffice was used to make 3D structures of key active constituents, and those of key targets were downloaded from the PDB database. PyMOL was used to remove water and phosphate from protein. AutoDock 1.5.6 was applied to convert the PDB format of the obtained file into the PDBQT format to find active pockets. Finally, the Vina script was run for docking. If the binding energy is less than 0, it means that the ligand and the receptor can spontaneously bind. At present, there is no uniform standard for the target screening of active constituents. According to the literature report [22], this study takes the binding energy less than or equal to −5.0 kJ/mol as the screening basis to evaluate the reliability of bioinformatics analysis and prediction.

### 2.8. Pharmacological Experiment Verification

#### 2.8.1. Reagents

The reagents used in this study include the following: quercetin (HY-18085, MCE, USA), luteolin (HY-N0162, MCE, USA), kaempferol (HY-14590, MCE, USA), cryptotanshinone (HY-N0174, MCE, USA), dexamethasone (HY-14648, MCE, USA), naringenin (S2394, Selleck, USA), Percoll (P4937-25ML, Sigma, USA), TRIzol (15596018, Thermo Scientific, USA), HiScript® III RT SuperMix for qPCR (+gDNA wiper) (R323-01, Vazyme, China), and Taq Pro Universal SYBR qPCR Master Mix (R323-01, Vazyme, China).

#### 2.8.2. Primer Sequences

The primer sequences used in this study include the following: IL-1*β*: F 5′-TGCCACCTTTTGACAGTGATG-3′ and R 5′-CAAAGGTTTGGAAGCAGCCC-3′; PTGS2: F 5′-CAAAGGTTTGGAAGCAGCCC-3′ and R 5′-CAAAGGTTTGGAAGCAGCCC-3′; HMOX1: F 5′-CAAAGGTTTGGAAGCAGCCC-3′ and R 5′-ACAAGACAGAAATACGAGACAGA-3′; STAT3: F 5′-ACAAGACAGAAATACGAGACAGA-3′ and R 5′-ACAAGACAGAAATACGAGACAGA-3′; CAT: F 5′-ACAAGACAGAAATACGAGACAGA-3′ and R 5′-ACAAGACAGAAATACGAGACAGA-3′; and GAPDH: F 5′-ACAAGACAGAAATACGAGACAGA-3′ and R 5′-ACAAGACAGAAATACGAGACAGA-3′.

#### 2.8.3. Separation of BMSCs

Mouse femoral heads were cut on a clean bench, and bone marrow was collected with PBS and injected into centrifuge tubes with 10 ml Percoll (P4937-25ML, Sigma, USA) solution slowly along the wall at a 1 : 1 volume ratio. After 30 min of 1500 rpm centrifugation, the ring-shaped white cloud layer cells were drawn at the junction of the liquid surface. The cells were resuspended with a medium and pipetted repeatedly to make a single-cell suspension. The cell suspensions were added with an osteogenic induction medium (10% FBS, 5 *μ*g/ml insulin, 0.1 *μ*M dexamethasone, 0.2 mM vitamin C, and 10 mM *β*-glycerophosphate) and cultured in an incubator. The medium was changed every 3 days, and differentiation lasted for 15 days.

#### 2.8.4. Grouping

There were 6 groups: the control group, quercetin group, luteolin group, kaempferol group, cryptotanshinone group, and naringenin group. Each group was first processed with 1 × 10^−6^ mol/l dexamethasone (HY-14648, MCE, USA) for 1 hour; each group was added with PBS 10 *μ*M and corresponding drug-processed cells. After 7-hour incubation, total RNA was extracted by TRIzol (15596018, Thermo Scientific, USA) for qPCR detection. Expression levels of target genes were determined by the 2-*ΔΔ*CT method. Expression levels were normalized according to the reference gene GAPDH. Data were presented as mean ± SD, and GraphPad Prism 6 was used for one-way ANOVA.

## 3. Results

### 3.1. Active Constituents and Targets of Gujiansan

After retrieving and screening the TCMSP and BATMAN-TCM databases, 232 active constituents were obtained. Among them, Radix Panacis Quinquefolii had 11, Heterophylly Falsestarwort Root 8, Radix Astragali 20, Cornu Cervi Pantotrichum 5, Notoginseng 8, Rhizoma Drynariae 18, Ganoderma 61, Radix Salviae Miltiorrhizae 65, Moghania 1, Litsea 3, Endothelium Corneum Gigeriae Galli 4, Herba Asari 8, Saffron 5, and Rhizoma Smilacis Glabrae 15. After deleting 87 duplicates, a total of 145 active constituents were obtained, and the corresponding gene name of their target proteins was queried, and a total of 395 active constituents-targets were obtained.

### 3.2. SANFH-Related Targets

**Figure 2:**
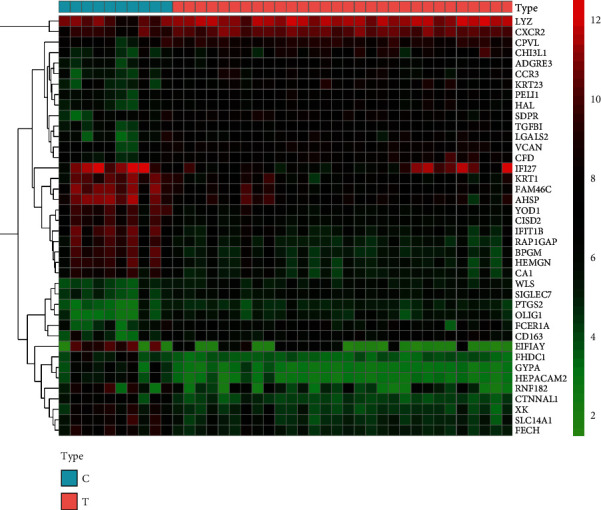
Differential gene heat map. Note: the first 10 columns are the genes of the healthy control group, and the last 30 columns are the genes of patients with SANFH. The color represents the degree of expression of this gene in different samples: green represents low expression, black represents medium expression, and red represents high expression.

The SANFH chip was analyzed with R language and other software, and a total of 1836 related targets of SANFH were obtained, including 1088 upregulated genes and 748 downregulated genes. The 20 genes with the most significant differences in upregulated and downregulated genes were selected, respectively, to draw the differential gene heat map (Figure 2).

### 3.3. “Active Constituent-Target” Network of Gujiansan in Treating SANFH

**Figure 3:**
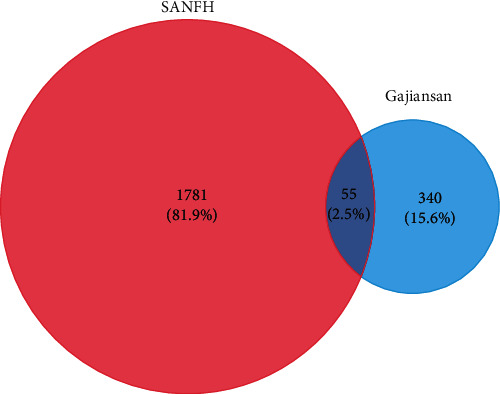
Venn diagram of potential targets of Gujiansan in treating SANFH.

**Figure 4:**
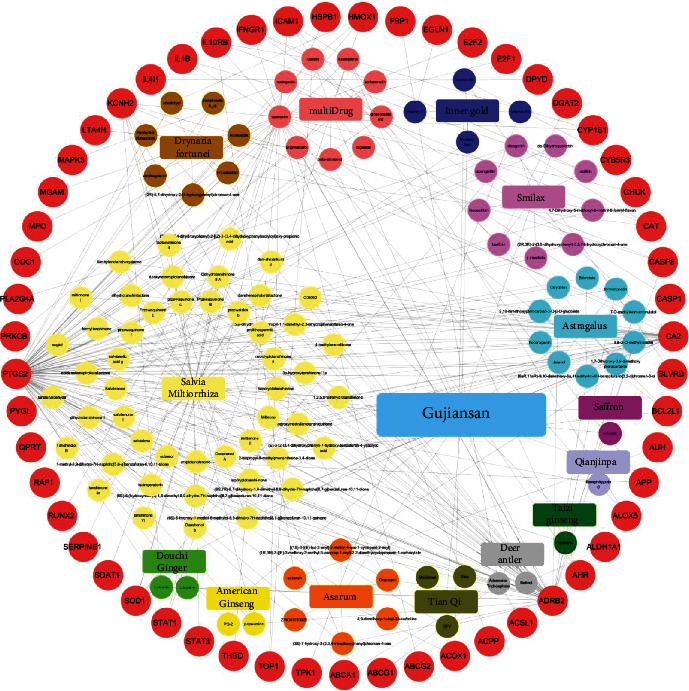
The “active ingredient-target” network of Gujiansan in the treatment of SANFH. Note: the circle in the outermost ring represents the target, the circle in the ring represents the active constituent, and the rectangle in the ring represents Chinese medicine.

The Venny platform was used to map and intersect the active constituents-targets of Gujiansan and the related targets of SANFH, and a total of 55 potential action targets of Gujiansan in the treatment of SANFH were obtained (Figure 3). The relationship between the active constituents and the intersecting targets was imported into Cytoscape to construct the “active constituent-target” network of Gujiansan in treating SANFH (Figure 4). The network consisted of 176 nodes (106 active constituent nodes, 55 target nodes, 14 TCM nodes, and 1 Gujiansan node) and 370 edges. The top five active constituents with moderate values in this network were quercetin, luteolin, kaempferol, cryptotanshinone, and naringenin, which were the key active constituents in this network and had important significance for the treatment of SANFH. The basic information on key active ingredients is shown in Table 1.

### 3.4. PPI Network

**Figure 5:**
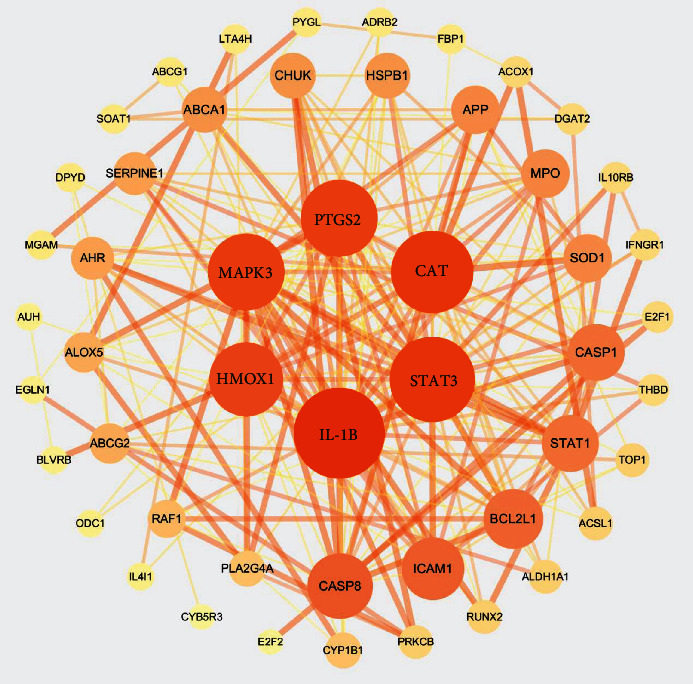
Protein interaction network. Note: nodes represent protein genes, while edges represent the interaction relationship between protein genes. The node size, color, and connection thickness are all topology parameters of a protein-protein interaction network. The degree value, as the number of connections between a node and other nodes in the network, is the most intuitive parameter to determine the “influence” of a node. The more connections a node has, the greater the degree value is, and the greater its influence will be.

Protein interaction is the basis of cell function, and it plays an important role in the regulation of physiology and pathology. In this study, the PPI network was constructed with the STRING database and Cytoscape software (Figure 5). There were 50 nodes and 206 edges in the graph. The top 6 protein genes screened according to the degree value were IL1B, STAT3, CAT, PTGS2, MAPK3, and HMOX1. These protein genes played a key role in the whole network as well as in the treatment of SANFH by Gujiansan. They may be the key targets of Gujiansan in the treatment of SANFH. The basic information on the key targets is shown in Table 2.

### 3.5. DAVID Enrichment Analysis

**Figure 6:**
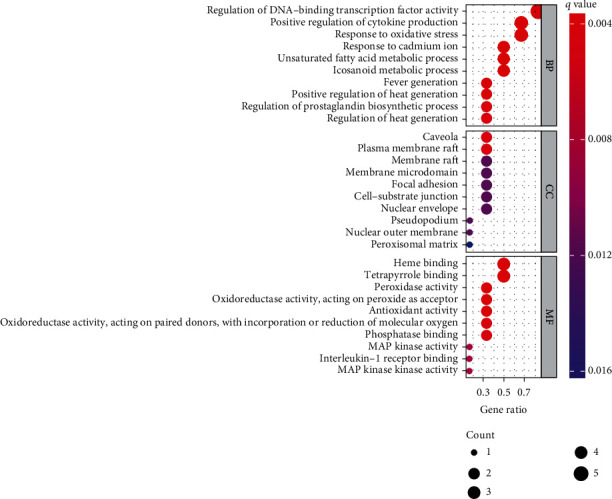
GO functional enrichment analysis.

**Figure 7:**
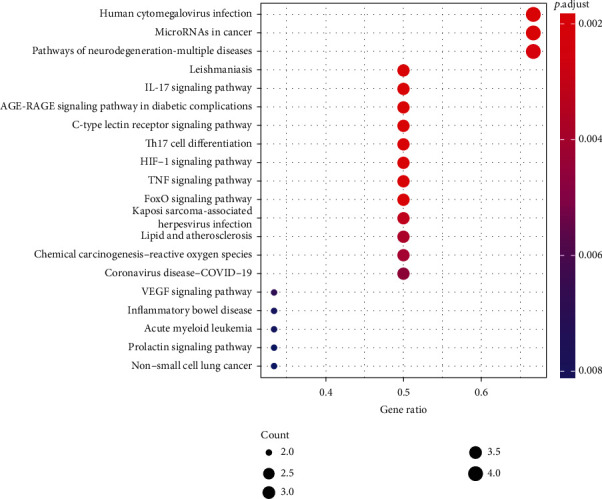
Enrichment analysis of the KEGG signaling pathway. Note: the abscissa is the enrichment fraction. A larger bubble indicates more genes enriched in this entry. A red bubble indicated more significant enrichment.

During the functional process of the action target of GO enrichment analysis, a total of 784 entries were identified, of which 734 entries represented biological processes (BP) and were mainly related to the regulation of DNA-binding transcription factor activity, positive regulation of cytokine production, response to oxidative stress, response to cadmium ion, and unsaturated fatty acid metabolic process. 19 of them represented cellular components (CC), mainly involving the caveola, plasma membrane raft, membrane raft, membrane microdomain, and focal adhesion; 31 represented molecular function (MF), which mainly referred to heme binding, tetrapyrrole binding, peroxidase activity, oxidoreductase activity, acting on peroxide as an acceptor, and antioxidant activity. BP, CC, and MF obtained from GO analysis results were closely related to the occurrence and development process of SANFH (Figure 6). A total of 53 entries were identified as targets for KEGG enrichment analysis, mainly involving the IL-17 signaling pathway, aging signaling pathway, C-type lectin receptor signaling pathway, HIF-1 signaling pathway, and TNF signaling pathway (Figure 7).

### 3.6. KEGG Relation Network

**Figure 8:**
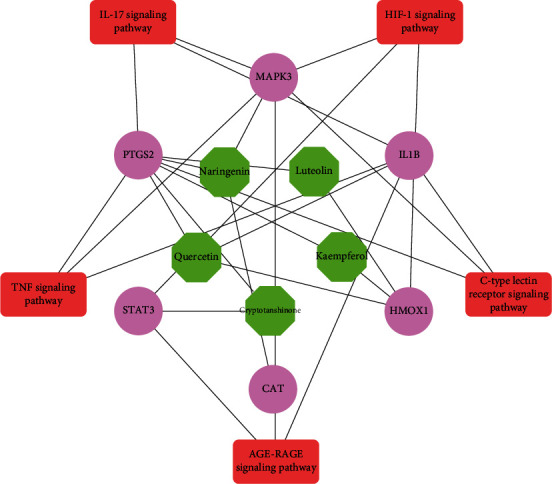
KEGG network diagram. Note: the hexagon in the inner circle represents key active constituents, the circle in the middle circle represents key targets, and the rectangle in the outer circle represents key signal pathways.

We visualized the relation network of “key active constituents-key targets-key signal pathways” of Gujiansan by Cytoscape software and constructed the “KEGG relation network diagram” (Figure 8). The results showed that Gujiansan functioned in treating SANFH through multiple targets and multiple signal pathways (Table 3).

### 3.7. Molecular Docking

**Figure 9:**
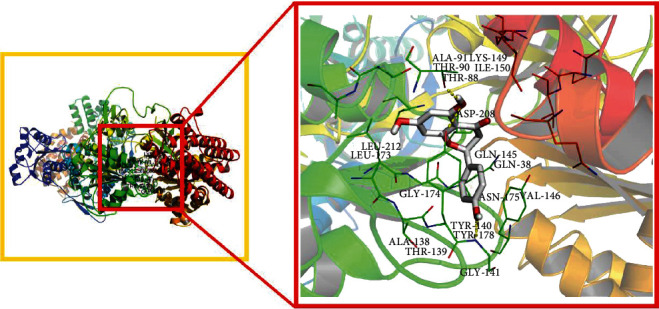
Molecular docking.

Generally, it is considered that the more stable the binding energy between the ligand and the receptor is, the lower the binding energy is, and the greater the possibility of action is. Based on the binding energy ≤ −5.0 kJ/mol, the predicted key active constituents and key targets were verified by molecular docking. The results showed that the affinity between the key active constituents of Gujiansan in treating SANFH and the key targets is far less than −5.0 kJ/mol, which indicates that they have good binding activity, which proves that the prediction of this study is reliable (Table 4). The affinity between naringenin and HMOX1 was the lowest, and the molecular docking display of naringenin is shown in Figure 9.

### 3.8. Main Constituents of Gujiansan Prevent GC-Induced Osteoblast Injury

**Figure 10:**
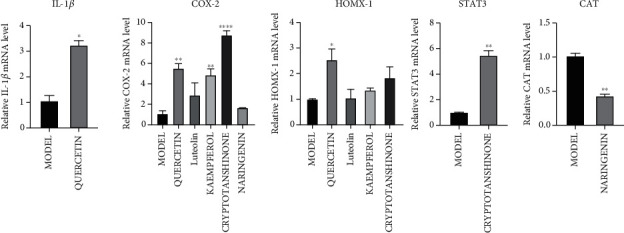
Effects of quercetin, luteolin, kaempferol, cryptotanshinone, and naringenin on the expression of related genes in osteoblasts induced by steroid-induced ischemic necrosis of the femoral head. Data were expressed as the mean ± SD. ^∗^*P* < 0.05 vs. the model group. ^∗∗^*P* < 0.01 vs. the model group. ^∗∗∗∗^*P* < 0.0001 vs. the model group.

This study detected five target genes (IL-1*β*, STAT3, CAT, PTGS2, and HMOX1) to further study the effects of quercetin, luteolin, kaempferol, cryptotanshinone, and naringenin on the expression of related genes in steroid-induced avascular necrosis of the femoral head. Compared with the model group, the IL-1*β* gene expression in the quercetin group was upregulated; the COX-2 gene expressions in the quercetin group, kaempferol group, and cryptotanshinone group were significantly upregulated; the expression of HMOX1 gene in the quercetin group was significantly upregulated; the expression of the STAT3 gene in the cryptotanshinone group was significantly upregulated; and the expression of CAT in the naringenin group was significantly downregulated (Figure 10).

## 4. Discussion

Traditional Chinese medicine also believes that blood supply disorder (blood stasis), bone damage (bone erosion), and lipid elevation (dampness and turbidity) are major pathological changes of SANFH, and the TCM compound, which can promote microcirculation, protect bone, and resist lipid, has always been an important nonsurgical therapy in Chinese guidelines for the treatment of ONFH [23]. At present, the commonly used western medicines for SANFH, such as bisphosphonate and iloprost, have adverse reactions. Relatively speaking, traditional Chinese medicines and proprietary Chinese medicines have relatively smaller adverse reactions and have shown beneficial results in preventing or delaying SANFH and maintaining physical functions [24–26]. In this study, 395 potential active constituents of Gujiansan were screened preliminarily, and 55 target genes were obtained from the targets of interaction between potential active constituents identified in Gujiansan and SANFH. A “single drug-active constituent-target” network was constructed to screen the main active constituents including quercetin, luteolin, kaempferol, cryptotanshinone, and naringenin. Quercetin and kaempferol can inhibit the enzyme activities of thrombin and FXa and the platelet functional response of the ROS-dependent signaling pathway, thus producing anticoagulant and antioxidant effects [27, 28]. In addition, quercetin, luteolin, and kaempferol can inhibit the expression of adipogenesis-related factors and lipid metabolism genes in 3T3-L1 preadipocytes, thereby reducing the accumulation of triglycerides and serving as antiadipogenesis [29–33]. Cryptotanshinone can inhibit the activity of the NF-*κ*B, AP-1 inflammatory transcription factor, and cyclooxygenase-2 enzyme in macrophages and play an anti-inflammatory role [34–37]. Naringenin inhibits the expression of lipid substances (such as TC and LDL) and apoptosis factors (such as caspase-3 and BAD), promotes the expression of cytokines such as AKT, RUNX2, Sp7, Notch, and alkaline phosphatase, and improves the imbalance of bone formation and absorption, thus alleviating the process of SANFH [38, 39].

**Figure 11:**
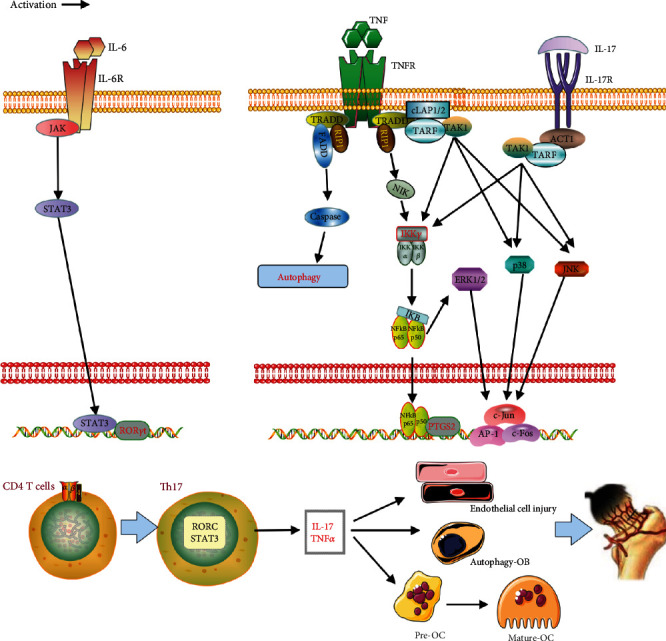
Action mechanism of IL-17 and TNF signaling pathways. Note: CD4 T cells on the left side of the figure activated ROR*γ*t expression by the IL-6/JAK/STAT3 signal and differentiated into Th17 cells. The secretion of IL-17 and TNF by Th17 cells, respectively, activates their respective signaling pathways. TNF on the right side of the figure can bind to TNFR and interact with the death domain (TRADD) to recruit factors such as TRAF, RIP1, FADD, and TAK1. RIP1 can directly activate NF-*κ*B. TAK1 can activate p38, JNK, and NF-*κ*B; IL-17 combines with IL-17R and then mediates the NF-*κ*B and MAPK signals by linking TRAF factors with ACT1 (TRAF3 Interacting Protein 2). These activated downstream factors regulate the transcriptional expression of AP-1, c-Fos, c-Jun, and PTGS2 genes in DNA and finally realize the upregulation of osteoclasts and negative regulation of osteoblasts and endothelial cells.

The six core target genes (IL1B, STAT3, CAT, PTGS2, MAPK3, and HMOX1) in the network were screened out by constructing PPIs. GO enrichment analysis indicated that many targets are involved in the regulation of DNA-binding transcription factor activity, positive regulation of cytokine production, and response to oxidative stress. The results of the KEGG pathway analysis suggested that several signaling pathways such as IL-17, AGE-RAGE, C-type lectin receptor, HIF-1, and TNF are mainly involved. (1) For the IL-17 signaling pathway, studies have shown that the secretion levels of IL-6, IL-17, IL-1*β*, TNF-*α*, and IL-33 in the serum of patients with SANFH were increased, and the high level of Th17 appeared [40–43]. CD4+ T cells can differentiate into Th17 cells when stimulated by IL-6, IL-1*β*, and IL-23. In this process, the binding of IL-6 to the IL-6 receptor activates JAK kinase phosphorylation, and the activated JAK then activates the STAT3 transcription factor. STAT3 induces the expression of Th17 cell-specific transcription factor ROR*γ*t in the nucleus and ultimately promotes CD4+ T cells to Th17 cells [39–42]. Activated Th17 mediates multiple downstream signals such as NF-*κ*B and MAP3K (JNK, p38, and ERK) by secreting IL-17 and ultimately promotes the gene expression of TNF-*α*, AP-1, and PTGS2/COX-2 [41–45] (Figure 11). (2) For the TNF signaling pathway, the TNF receptor (TNFR) is expressed in a variety of human cells (such as macrophages, chondrocytes, osteoclasts, osteoblasts, and endothelial cells). TNF binding to TNFR activates TRADD (TNFR-associated death domain), which in turn recruits RIP (receptor-interacting protein) and FADD (Fas-associated death domain) to activate downstream MAP3K and caspase signals, respectively [46]. Studies in osteoclasts show that TNF-mediated MAP3K increases osteoclast survival and the expression of both mRNA and protein. In addition, TNF and RANKL can synergistically induce the differentiation of bone marrow-derived macrophages (BMMs) into osteoclasts [47–49]. However, TNF in chondrocytes, osteoblasts, and endothelial cells functioned in inhibiting cell differentiation and proliferation and even promoting apoptosis [50–52] (Figure 11). (3) The HIF-1 signaling pathway is the main response mechanism of the systemic and cellular steady-state response to hypoxia. The HIF-1 signal activates the transcription of many genes through oxygen-regulated *α*-subunits and constitutively expressed *β*-subunits, such as activating HMOX1 to promote heme catabolism, activating VEGF to promote angiogenesis, and activating the BNIP3 autophagy factor to reduce apoptosis. HIF-1*α* expression was decreased in the necrotic bone tissue of SANFH, and HIF-1*α* compensatory expression was upregulated in early bone tissue under hypoxic or anoxic conditions. However, with the progression of the disease, the imbalance of energy metabolism in necrotic bone tissue was aggravated, and the expression levels of HIF-1*α*, VEGF, HMOX1, and BNIP3 were decreased, resulting in the decreased differentiation and apoptosis of vascular endothelial cells [53, 54]. (4) For the AGE-RAGE signaling pathway, under the action of GC, advanced glycation end products (AGE) and ROS accumulation occur in bone tissues and blood vessels of necrotic bone regions [55]. In vitro aging-related studies reveal that AGE can induce apoptosis of long bone cells by binding to RAGE and activating ERK1/2, p38, and STAT3 signaling pathways. The AGE-RAGE signal can also induce vascular endothelial cell injury and change vascular permeability by activating ERK and p38 [56, 57]. (5) For the C-type lectin receptor signaling pathway, it is researched that necrotic bone cells of patients with osteonecrosis exhibited highly expressed C-type lectin (Mincle). After binding to the C-type lectin receptor, Mincle triggered osteoclastogenesis by activating the calcium signaling pathway of ITAM, which activates osteoclasts and intensifies bone resorption [58]. The analysis of these signaling pathways also reflected the process of GO enrichment and analysis of cell activity.

Molecular docking results showed that quercetin, luteolin, kaempferol, cryptotanshinone, and naringenin interacted with the six core target genes (IL1B, STAT3, CAT, PTGS2, MAPK3, and HMOX1). The six core target genes also participated in the conduction of the five signaling pathways. Therefore, this may indicate the overall effectiveness and diversity of constituents of Gujiansan in intervening with SANFH and offer insights into the occurrence and development of other osteoarticular-related inflammatory diseases.

**Figure 12:**
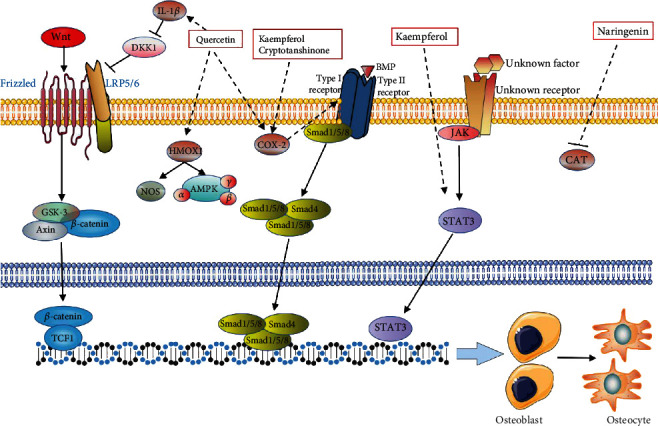
The mechanism of the main components of Gujiansan in regulating osteoblasts.

In the osteoblast experiments in this study, the results of qRT-PCR indicated that quercetin could significantly promote the upregulation of IL-1*β* gene expression in SANFH osteoblasts; studies also showed that IL-1*β* could inhibit the production of DKK1, thereby promoting the activation of Wnt signaling [59]. The activation of Wnt signaling could enhance the formation of MSC cartilage [60–62]. Therefore, it suggests that quercetin in this study may activate the Wnt signaling pathway by upregulating IL-1*β* gene expression in osteoblasts and finally promote cartilage formation. Quercetin, kaempferol, and cryptotanshinone could also significantly promote the upregulation of COX-2 gene expression in SANFH osteoblasts; COX-2 was essential for the osteogenic differentiation of MSCs. Studies have shown that COX-2 was induced in the early stages of bone repair and was produced in large amounts in local areas. COX-2 could induce and/or synergize with BMPs to increase RUNX2 and osterix, while these two factors were two important transcription factors required for endochondral and intramembranous bone formation [63]. Quercetin, kaempferol, and cryptotanshinone could promote the osteogenic differentiation of SANFH by upregulating the expression of the COX-2 genes, and quercetin could also upregulate the expression of the HMOX1 gene in steroid-induced osteonecrosis of the femoral head. Studies have shown that the expression of the HMOX1 gene could increase pAMPK and eNOS to promote osteoblast differentiation [64], which was consistent with the results of high expression of the HMOX1 gene in SANFH osteoblasts in this research. This study suggested that kaempferol was able to upregulate the expression of the STAT3 gene in osteoblasts. STAT3 as a member of the Janus Kinase-Signal Transducer and Activator of Transcription (JAK-STAT) protein family could regulate growth factors and various cytokines and play a key role in regulating cell proliferation, differentiation, migration, apoptosis, and survival. Studies have shown that STAT3 could drive osteoblast differentiation and bone formation, while induced loss of STAT3 in osteoblasts would impair bone remodeling and reduce the bone mass of adult mice [65] (Figure 12).

Catalase (CAT) was a key antioxidant enzyme in the body's defense against oxidative stress, which could convert reactive oxygen species H_2_O_2_ into water and oxygen, thereby reducing the toxic effects of hydrogen peroxide. Studies have shown that high doses of GC could induce osteoblast H_2_O_2_, generate and reduce CAT expression, and ultimately produce cytotoxicity [66, 67]. Low-dose GC (DEX (10-8 M)) would promote autophagy and cell activity of osteoblasts while inhibiting CAT [68]. Fernandes et al. showed that reactive oxygen species were involved in integrin signaling, which in turn mediated osteoblast adhesion/diffusion, after which osteoblast antioxidants gradually decomposed reactive oxygen species [69]. Naringenin was considered to be a drug with antioxidant effects. Naringenin could counteract the oxidative damage to the liver caused by Adriamycin by supplementing the reduced CAT [70, 71]. However, Kuang et al. showed that naringenin promoted GC-induced osteoblast proliferation [38]. The results of this study showed that the expression of the CAT gene was high in DEX-induced osteoblasts, while the expression of CAT was downregulated by naringenin, which led to a controversial conclusion. First, CAT in GC-induced osteoblasts mediated other life processes. Second, naringenin had antioxidant effects and could positively regulate osteoblasts under the action of GC; but whether naringenin affected osteoblasts through antioxidant regulation remains to be further studied.

It was found that Gujiansan could promote osteoblast differentiation by regulating multiple genes, and an interesting phenomenon was observed that the Chinese herbal compound reduced the inflammatory factors (such as TNF-*α* and IL-6) in the blood of SANFH rats in in vitro expression and elevated some inflammatory factors (such as IL-1*β* and COX-2) of osteoblasts to promote osteoblast differentiation. This antagonistic dual regulatory effect was the great potential of traditional Chinese medicine in the treatment of SANFH.

The insufficiency of this study was that its prediction of the pharmacological mechanism of Gujiansan in the intervention of SANFH was based on the previous research findings of the team and the existing database. In order to ensure the reliability and rationality of the prediction results, further experimental verification was required. Certain limitations exist though the results of network pharmacology analysis and molecular docking analysis indicated the characteristics of Gujiansan in the treatment of SANFH. This study further elaborated on the pathogenesis of SANFH and brought new treatment strategies for the prevention and treatment of SANFH.

## Figures and Tables

**Table 1:** Basic information on key active ingredients.

Active ingredient	Degree	Traditional Chinese medicine	Structure	Target
Quercetin	30	Astragalus, Tian Qi, Smilax, Saffron	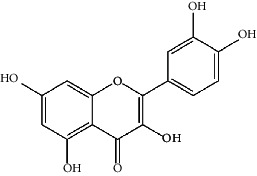	PTGS2, KCNH2, ADRB2, BCL2L1, IL10RB, ODC1, CASP8, TOP1, RAF1, SOD1, STAT1, HMOX1, ICAM1, IL1B, PRKCB, HSPB1, MGAM, CYP1B1, THBD, SERPINE1, IFNGR1, ALOX5, MPO, ABCG2, AHR, CHUK, RUNX2, E2F1, E2F2, ACPP
Luteolin	8	Salvia Miltiorrhiza, Drynaria Fortunei, Taizi Ginseng	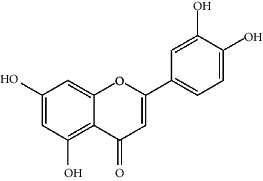	PTGS2, BCL2L1, IL10RB, TOP1, APP, HMOX1, ICAM1, IFNGR1
Kaempferol	7	Drynaria Fortunei, Astragalus, Saffron, Asarum	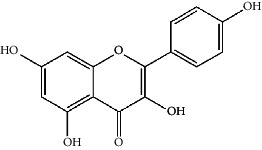	PTGS2, STAT1, HMOX1, ICAM1, CYP1B1, ALOX5, AHR
Cryptotanshinone	6	Salvia Miltiorrhiza	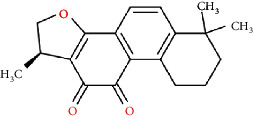	PTGS2, CA2, ADRB2, STAT3, BCL2L1, APP
Naringenin	5	Drynaria Fortunei, Smilax	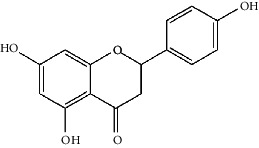	PTGS2, MAPK3, SOD1, CAT, SOAT1

**Table 2:** The basic information on the key targets.

No.	Target	Related description	Degree
1	IL1B	Interleukin 1 beta	26
2	STAT3	Signal transducer and activator of transcription 3	24
3	CAT	Catalase	23
4	PTGS2	Prostaglandin-endoperoxide synthase 2	21
5	MAPK3	Mitogen-activated protein kinase 3	21
6	HMOX1	Heme oxygenase-1	20

**Table 3:** KEGG network.

Key active ingredients	Key target	Key signaling pathway
Quercetin	IL1B	IL-17 signaling pathway, AGE-RAGE signaling pathway, C-type lectin receptor signaling pathway, TNF signaling pathway
	PTGS2	IL-17 signaling pathway, C-type lectin receptor signaling pathway, TNF signaling pathway
	HMOX1	HIF-1 signaling pathway
Luteolin	PTGS2	IL-17 signaling pathway, C-type lectin receptor signaling pathway, TNF signaling pathway
	HMOX1	HIF-1 signaling pathway
Kaempferol	PTGS2	IL-17 signaling pathway, C-type lectin receptor signaling pathway, TNF signaling pathway
	HMOX1	HIF-1 signaling pathway
Cryptotanshinone	STAT3	AGE-RAGE signaling pathway, HIF-1 signaling pathway
	PTGS2	IL-17 signaling pathway, C-type lectin receptor signaling pathway, TNF signaling pathway
Naringenin	CAT	
	PTGS2	IL-17 signaling pathway, C-type lectin receptor signaling pathway, TNF signaling pathway
	MAPK3	IL-17 signaling pathway, AGE-RAGE signaling pathway, C-type lectin receptor signaling pathway, HIF-1 signaling pathway, TNF signaling pathway

**Table 4:** Binding energy of key active components and key targets.

Compound	Binding energy (kJ·mol^−1^)					
	IL1B	STAT3	CAT	PTGS2	MAPK3	HMOX1
Quercetin	-23.85	-32.64	-36.82	-33.47	-35.56	-35.98
Luteolin	-26.36	-28.87	-36.82	-33.05	-35.98	-36.82
Kaempferol	-23.43	-28.87	-35.56	-31.38	-34.73	-34.73
Cryptotanshinone	-23.01	-28.45	-23.85	-28.87	-31.38	-23.85
Naringenin	-23.01	-29.71	-27.61	-33.89	-28.87	-37.24

## Data Availability

The data used to support the findings of this study are available from the corresponding author upon request.

## Supplementary Materials

Supplementary Materials Table 5: involved database in the study. Table 6: involved related analysis platform in the study.
