# Supplementary material for: Network-Based Pharmacology and Bioinformatics Study on the Mechanism of Action of Gujiansan in the Treatment of Steroid-Induced Avascular Necrosis of the Femoral Head
Source: Biomed Res Int. 2022 Jul 23;2022:8080679. doi: 10.1155/2022/8080679 (PMC9338865; doi:10.1155/2022/8080679)
Supplement: Supplementary Materials — Table 5: involved database in the study. Table 6: involved related analysis platform in the study. [file 8080679.f1.docx]

| **NO** | **Name** | **Website** | **Version** |
| --- | --- | --- | --- |
| 1 | Traditional Chinese Medicine Systems Pharmacology Database and Analysis Platform (TCMSP) | Web:https://old.tcmsp-e.com/tcmsp.php | Ver.:2.3 |
| 2 | a Bioinformatics Analysis Tool of Molecular mechanism of Traditional Chinese Medicine(BATMAN-TCM) | Web:http://bionet.ncpsb.org.cn/batman-tcm/ | Ver.:Updated on July 29, 2020 |
| 3 | UniProt Database | Web:https://www.uniprot.org | Ver.:updated on February 10, 2021 |
| 4 | Gene Expression Omnibus database（GEO） | Web:https://www.ncbi.nlm.nih.gov/geo/ | Ver.:Updated on  November 1, 2021 |
| 5 | Protein Interaction Database （STRING） | Web:https://string-db.org/ | Ver.:11.0 |
| 6 | Biological Information Annotation Database（DAVID） | Web:http://david.ncifcrf.gov. | Ver.:6.8 |
| 7 | RCSB PDB database | Web:http://www.rcsb.org | Ver.:updated on May 05, 2020 |

**Table 5 Involved database in the study**

| **NO** | **Name** | **Website** | **Version** |
| --- | --- | --- | --- |
| 1 | perl language | Web:https://www.perl.org/ | Ver.:5.30.0.1 |
| 2 | R language | Web:https://www.r-project.org/ | Ver.:4.1.1 |
| 3 | Venny | Web:http://bioinfogp.cnb.csic.es/tools/venny | Ver.:2.1.0 |
| 4 | Cytoscape software | Web:http://www.cytoscape.org/ | Ver.:3.7.2 |
| 5 | PyMOL software | Web:https://pymol.org/2 | Ver.:1.7.2.1 |
| 6 | AutoDuckTools software | Web:http://autodock.scripps.edu/resources/adt | Ver.:1.5.6 |
| 7 | Autodock vina software | Web:http://vina.scripps.edu | Ver.:1.1.2 |

**Table 6 Involved related analysis platform in the study**
